# What is the clinical course of transient synovitis in children: a systematic review of the literature

**DOI:** 10.1186/2045-709X-21-39

**Published:** 2013-11-14

**Authors:** Sylvana S Asche, Rogier M van Rijn, Johannes HJM Bessems, Marjolein Krul, Sita MA Bierma-Zeinstra

**Affiliations:** 1 Department of General Practice, Erasmus MC-University Medical Centre Rotterdam, PO Box 2040, 3000 CA, Rotterdam, The Netherlands; 2 Department of Orthopedic Surgery, Erasmus MC-Sophia Children’s Hospital Rotterdam, Dr. Molewaterplein 60, 3015 GJ, Rotterdam, The Netherlands; 3 Department of Public Health, Erasmus MC-University Medical Centre Rotterdam, PO Box 2040, 3000 CA, Rotterdam, The Netherlands

**Keywords:** Child, Hip, Transient synovitis, Coxitis fugax, Legg-Perthes’ disease, Course, Prognosis, Follow-up

## Abstract

**Background:**

Transient synovitis of the hip (TS) is considered to be a self-limiting disease in childhood. However, because the etiology is unclear and some cases precede Legg-Perthes’ disease, data on follow-up are important. Our aim was to summarize the knowledge on the clinical course of TS in children.

**Methods:**

The study design was a systematic review and a literature search was conducted in Medline and Embase. Studies describing short and/or long-term follow-up of TS in children were included. Case reports, reviews and studies describing traumatic hip pain were excluded. Study quality was scored and data extraction was performed. The main outcome measures were short-term and long-term clinical course, and recurrence of symptoms.

**Results:**

A total of 25 studies were included of which 14 were of high quality. At two-week follow-up, almost all children with TS were symptom free. Those with symptoms persisting for over one month were more prone to develop other hip pathology, such as Legg-Perthes’ disease. The recurrence rate of TS ranged from 0–26.3%. At long-term follow-up, 0-10% of the children diagnosed with TS developed Legg-Perthes’ disease. Hip pain after intensive physical effort and limited range of motion of the hip at long-term follow-up was reported in 12-28% and in 0-18% of the children, respectively.

**Conclusions:**

The majority of the studies indicate that children with TS recover within two weeks; recurrence was seen in 0-26% of the cases. Children with TS should be followed at least six months to increase the likelihood of not missing Legg-Perthes’ disease.

## Background

Transient synovitis of the hip (TS) in children is a relatively common disorder with an average annual incidence of 0.2% in the general population [1]. There is a male-to-female ratio of slightly more than 2:1. The disease typically presents at a mean age of 6 (3–8) year [1].

TS is characterized by pain and limited motion of the hip [1]. This hip pain and limited range of movement is caused by reactive effusion, leading to a position of the hip in flexion and external rotation [2]. TS is confirmed by excluding potentially more severe causes for hip symptoms, such as septic arthritis, osteomyelitis, Legg-Perthes’ disease, juvenile idiopathic arthritis, fractures and tumors [1]. These conditions can be verified by imaging and laboratory investigations [3]. However, the mainstay of diagnosis for TS remains that of clinical findings [3]. For detection of hip effusion, ultrasound is considered to be the best non-invasive technique [2]. The etiology of TS remains largely unknown. Infections of the upper respiratory tract may be a predisposing factor, although identifiable causes or agents have not yet been found [4]. Treatment generally consists of rest and anti-inflammatory agents [5].

In many countries, the majority of children with non-traumatic hip symptoms will initially present to the general practitioner (GP). Krul *et al.*[6] reported an incidence rate for TS in general practice of 76.2 per 100,000 person-years [6]. According to Vijlbrief *et al.*[7], GPs prefer to adopt a wait-and-see approach to TS rather than referring patients for additional diagnostics [7]. However, TS has been associated with the risk of developing other diseases, such as Legg-Perthes’ disease [1].

Increased understanding of the short and long-term course of this disease will help medical doctors to better determine follow-up policy and better inform patients about the prognosis. Early recognition of subsequent disorders may also lead to timely referral and more optimal treatment. Therefore, this study presents a systematic review of the literature on TS, focusing on its short-term symptomatic course, long-term clinical course, and recurrences.

## Methods

### Searches

A literature search using Medline and Embase was performed from Januari 1966 (Medline) and Januari 1947 (Embase) till September 2013. Medical subject headings (mesh) terms and corresponding entry terms for the diagnosis, age group and outcome were used (Additional file 1: Table S1).

### Study inclusion and exclusion criteria

Studies describing recurrence or clinical follow-up (either on the short or long-term) of TS in children were included. Studies describing children with traumatic hip problems were excluded, as were reviews and case reports. Randomized controlled trials, and cohort and case–control studies were included. Relevant articles in English, German, French, Swedish, Norwegian, Danish and Dutch were included. The abstract of the article had to be available in order to select relevant studies. First, two reviewers (SA, RvR) selected relevant articles by title and abstract. All articles selected by either reviewer were ordered in full text. One reviewer (SA) made the final selection by reading the full text of the selected articles. In case of doubt, a second reviewer (SB) read the full text to ensure that no studies were excluded unjustly.

### Study quality assessment

Three reviewers assessed the quality of the selected articles independently. One reviewer (SA) assessed the quality of all studies and two reviewers (SB, JB) each assessed the quality of half of the studies. Seven criteria were used, which were rated as positive, negative or inconclusive. These criteria were based on the quality assessment used by van Rijn *et al.*[8] and adjusted for the purpose of investigating TS. Disagreements were resolved by consensus. The initial agreement was measured by using the Kappa value (k). Agreement is considered poor if k ≤ 0.20; fair if 0.21≤ k ≤ 0.40; moderate if 0.41 ≤ k ≤ 0.60; substantial if 0.61 ≤ k ≤ 0.80; good if k > 0.80 [9]. Summing the positive criteria, a quality score was calculated ranging from 0 to 7 points. Studies with five or more positive criteria were considered to be of 'high quality’.

### Data extraction strategy

One reviewer (SA) performed data extraction for all the studies, which was subsequently checked by two other reviewers (RvR and MK) each checking half of the studies. Data extraction included number of patients with TS; characteristics of patients with TS (age, male/female ratio, primary or secondary care, duration of symptoms prior to admission); study design; tests done before diagnosing TS; treatment for TS; length of follow-up period; and outcomes (short-term symptomatic course, recurrence, long-term course). Disagreements were resolved by consensus.

### Data synthesis and presentation

Included articles were classified in three groups based on their outcome: short-term symptomatic course, recurrence and/or long-term course. Short-term symptomatic course implied clinical follow-up < 3 months. Studies classified as *recurrence* reported a recurrence rate and/or clinical course of patients with recurrent TS. Long-term course was defined as clinical course of TS > 3 months; this also included the development of other diseases subsequent to TS.

## Results

### Review statistics

**Figure 1:**
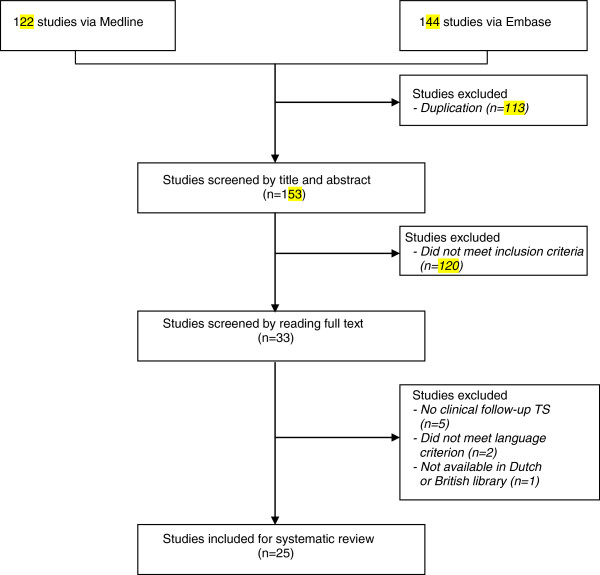
Flow chart of the selection process.

After removal of duplicates, our search strategy resulted in 153 potentially relevant studies. Of these, 25 met the inclusion criteria (Figure 1). Study selection by title and abstract reached an initial agreement of 90.4%; after further consultation 100% agreement was achieved. Details of the study characteristics are listed in Table 1. We found no randomised clinical trials or case–control studies. Fifteen cohort studies [3,4,10-22] were prospective and 10 [1,2,23-30] were retrospective. All studies were written in English except for three, of which two were written in German [11,21] and one in French [26]. The number of patients with TS ranged from 13 [17] to 475 [24]. Patients with TS were followed either after admission to hospital or as outpatients.

**Table 1:** Study characteristics

**Source**	**No. TS**^**a**^	**Prospective/ Retrospective**	**Patient characteristics**	**Tests before diagnosing TS**^**b**^	**Age mean ± SD (range)**	**M/F ratio**	**Treatment**	**Follow-up period mean (range)**
Uziel 2006 [30]	39	Retrospective	Patients with at least 2 separate episodes of transient synovitis, at least 2 months apart, seen by pediatric rheumatologists.	- Lab: ESR, WCC, ANA, RF, FMF, HLA-B27	6 ± 2.6 years	2:1	13 pt: NSAIDs, 5 pt: skin traction.	4.2 ± 2.5 years
				- Plain radiographs of the hip, ultrasound, MRI, technetium bone scans				
Skinner 2002 [3]	25	Prospective	Patients with TS with proven hip joint effusions detected on ultrasound, managed in the accident and emergency department as outpatients. The median duration prior to presentation was 2 days.	- History, full clinical examination, temperature	6 (4–11) years	4:1	Rest at home, paracetamol.	1–2 weeks
				- Lab: FBC, ESR				
				- Ultrasound, radiography of the hip				
Kermond 2002 [18]	36	Prospective	Patients with clinical diagnosis of TS with a median duration of symptoms prior to presentation of 1 day.	- Clinical examination of the hip	1–11 years	4.1:1	17 pt. ibuprofen/paracetamol19 pt. placebo/paracetamol.	Time till symptom free
				- Lab: full blood examination, ESR, CRP				
				- Radiography of the hip in AP + frog leg lateral views				
Fischer 1999 [4]	65	Prospective	Patients with a history of atraumatic limp who presented to a paediatric accident and emergency department either by self referral or from their general practitioner, with a median duration of symptoms of 1 day at presentation.	- History, full clinical examination, temperature	4.35 years^c^	1.7:1^c^	Unclear	18–21 months
				- Lab: FBC, ESR				
				- Ultrasound, plain radiographs of the hip				
Mattick 1999 [27]	103	Retrospective	Patient with hip pain or suspected hip pathology presenting to a Children’s Hospital.	- Range of movement of the hip, temperature	0.9-15 years (median age of 5 years)	3:1	Rest at home.	7 years
				- Lab: ESR, WBC				
				- Radiography of the hip				
Eggl 1999 [12]	106 hips	Prospective	Patients with hip pain and a preliminary diagnosis of TS referred to an outpatient clinic.	- History, clinical examination	5.7 (1.8–10.1) years	2.8:1	Bed rest, positioning hip in flexion and oral application of naproxen.	42 days
				- Lab: CRP, ESR, blood count, ASLO, electrophoresis				
				- Ultrasound				
De Pellegrin 1997 [11]	100	Prospective	Patient with hip pain presenting to the orthopedic department with duration of symptoms prior to admission of 1 to 60 days.	- History	4.14 ± 2.2 (1–10) years	1.8:1	Positioning hip in flexion/ abduction and external rotation and analgesics.	Unclear
				- Lab: full blood examination				
				- Ultrasound, scintigraphy, radiography of the hip, MRI				
Kesteris 1996 [19]	21	Prospective	Patients with TS admitted to the Department of Orthopedics, treated in two consecutive groups. The mean duration of symptoms before admission was 2.6 days.	Unclear	7.1 (4–12) years	9.5:1	12 pt: arthrocentesis,	6–12 months
							9 pt: no arthrocentesis	
							All children were kept in bed in the supine position with the hips in a few degrees of flexion.	
Keenan 1996 [17]	13	Prospective	Children with a recurrent or prolonged irritable hip syndrome, defined as those with symptoms >1 month or in whom symptoms recurred within 1 month after initial resolution.	- Clinical examination of the hip	(4–8 years)	Not given	Restriction sporting activities, bed rest with positioning hip in flexion/abduction and external rotation.	3 months
				- Radiography of the hip in Lauenstein + frog lateral views, ultrasound				
Taylor 1995 [29]	426	Retrospective	Patients with an irritable hip admitted to hospital with a mean duration of symptoms before admission ranging from 3.7 to 4.9 days.	- History, temperature, range of movement	A: 5.7 years	A: 2.3:1	Unclear	Unclear
						B: 2.4:1		
					B: 6.6 years	C: 2:1		
				- Lab: FBC, ESR				
					C: 6.4 years^d^			
				- Radiography of the hip in AP + frog lateral views, bone isotope scans, MRI				
Gopakumar 1992 [2]	181	Retrospective	Patients with suspected irritable hip syndrome admitted to hospital with a mean duration of symptoms prior to presentation of 3 days.	- Clinical examination of the hip	7 (0.9–14) years	2:1	Bed rest followed by early mobilization.	7 (6–24) months
				- Lab: WBC, ESR				
				- Radiography of the hip in AP, ultrasound, bone isotope scan				
Terjesen 1991 [22]	58	Prospective	Patients with TS treated in the Department of Orthopedic Surgery with a mean duration of symptoms prior to admission of 3 days.	- Ultrasound, radiography of the hip in AP	5.8 (2–15) years	3.1:1	Bed rest, bilateral skin traction and moderate flexion of the hip.	11 (6–26) weeks
Briggs 1990 [23]	286	Retrospective	Children with TS presented to a Children’s Hospital.	- Clinical examination	(2–13 years)	2:1	Bed rest with or without traction applied to the affected leg with the hip in extension.	Unclear
				- Lab: FBC, ESR, serum urea, electrolyte estimations, bacteriological investigations: by throat swabs, urine cultures and blood cultures, agglutinins against Salmonella, brucellosis, viral antibodies, Paul Beunnell test, RA Latex and Rose-Waaler titers, antinuclear factor, Tine test or Mantoux test.				
				- Radiography of the hip in AP and Lauenstein views				
Hasegawa 1988 [14]	55	Prospective	Children with the clinical diagnosis TS admitted for examination at hospital, with duration of symptoms prior to examination of 18 days.	- Range of movement	7 (2–13) years	2.4:1	Unclear	4–9 months
				- Radiography of the hip in AP + Lauenstein views				
Kallio 1988 [16]	109	Prospective	Children with TS admitted to hospital, with duration of symptoms prior to admission of 3 days in 75% of the cases.	- Clinical examination	5.6 years	2:1	Unclear	1 year
				- Hematologic and serologic examinations, bacterial cultures from the throat, blood, urine and synovial fluid				
				- Radiography of the hip in AP + Lauenstein views, ultrasound, hip aspiration with intraarticular pressure				
Landin 1987 [1]	275	Retrospective	Children with TS admitted to hospital or treated as outpatients with a 1 to 7 day history of symptoms prior to treatment.	- Range of movement, temperature	(1–13 years)	2.6:1	Skin traction with the affected hip placed in extension and/or bed rest.	3 months–9 years
				- Lab: Hb, WBC, antistreptolysin, CRP, antibodies against Yersinia enterocolitica and Shigella, bacterial cultures from throat swab and urine				
				- Radiography of the hip in AP + frog lateral views				
Egund 1987 [13]	70	Prospective	Children with TS admitted to the Department of Diagnostic Radiology and Orthopedics.	- History, clinical findings	6 (1–12) years	2.7:1	Unclear	6.5 (5–9) months
				- Radiography of the hip in AP + Lauenstein, CT, ultrasound				
Kallio 1986 [15]	119	Prospective	Children with TS seen at a Children’s Hospital.	- Clinical examination	Not given	Not given	Bed rest.	1 year
				- Hematology, serology and bacterial culture from throat, blood, urine and synovial fluid				
				- Radiography of the hip in AP + Lauenstein views, ultrasound, hip aspiration				
Haueisen 1986 [24]	475	Retrospective	Children with TS who have been hospitalized.	- Physical examination, temperature	6.2 (0.8–18) years	2.2:1	Bed rest, Buck’s traction, antibiotics, spica casting.	6 months–4 years
				- Lab: RF, ANA, WBC, ESR				
				- Radiography of the hip, hip aspiration				
Mukamel 1985 [20]	41	Prospective	Children with TS followed as outpatients.	- History, physical examination	Average of 3.8 (1.3–12.5) years	3:1	Bed rest and analgesics.	9.3 months (3 months–3 years)
				- Lab: ESR, WBC				
				- Radiography of the hip				
Illingworth 1983 [25]	54	Retrospective	Children with a first episode of TS or with a recurrence of TS who attended hospital.	- Range of hip movement	6.4 (2–12) years	4.4:1	Unclear	Unclear
				- Lab: ESR, WBC, CRP, ASO, RF, bacterial culture from throat and stool, monospot test for infectious mononucleoisis				
				- Radiography of the hip				
Sharwood 1981 [28]	101	Retrospective	Children with TS admitted to a Children’s Hospital with a period of symptoms prior to admission <7 days in 82% of the cases.	- Clinical examination of the gait, range of hip movement and leg length	5.6	Unclear	Unclear	Average of 8.2 (5–15) years
				- Lab: ESR, WBC				
				- Radiography of the hips in AP + frog lateral positions.				
Calver 1981 [10]	50	Prospective	Children with TS presented at a Children’s Hospital with a mean duration of symptoms prior to admission of 4.5 days.	- Range of hip movement	6.1 (3–12) years	2:1	Bed rest and skin traction followed by slow mobilization.	1 year
				- Lab: blood count, blood film, ESR, RA latex agglutination, Widal and anti-staphylococcal titre, bacterial cultures from the throat and urine				
				- Radiography of the hip in AP + frog lateral views, radioisotope scans.				
Mallet 1981 [26]	38	Retrospective	Children admitted to hospital, with duration of symptoms prior to admission between 0 and 45 days.	- Radiography of the hip	6 (2–13) years	1.1:1	Skin traction, plaster.	7 (2–20) years
Stock 1977 [21]	34	Prospective	Children treated for TS at the Orthopedic Department, with duration of symptoms prior to admission mostly less than 1 week.	- History, physical examination	7.9 (4–14) years	1.6:1	Bed rest, relievement of hip, antibiotics, plaster, Tanderil, remedial therapy.	4.3 (0.5–15) years
				- Lab: WBC, AST, CRP				
				- Radiography of the hip				

^a^Number of children with transient synovitis of the hip included in the study; ^b^Not all tests were performed on every patient; some tests were only done if certain diseases were suspected; ^c^Given of the whole studied group of 243 children with a limp; ^d^A: No recurrence B: 1 recurrence C: >1 recurrence; TS: children with transient synovitis of the hip; ESR: erythrocyte sedimentation rate; Hb: haemoglobin concentration; WBC: white blood cell count; ANA: antinuclear antibodies; RF: rheumatoid factor; FMF: familial Mediterranean fever; FBC: full blood count; CRP: C-reactive protein; ASO: antistreptolysin O titre.

All studies included patients treated for TS, of which three studies [17,29,30] had a specific target group. One study [30] focused on patients with recurrences of TS, defined as at least two separate episodes of TS at least two months apart. One study [17] included patients with recurrent or prolonged TS, defined as recurrence of symptoms within one month after initial resolution, or symptoms that lasted more than one month. Another study [29] compared patients with no recurrence of TS with patients who had one or more recurrence(s).

### Study quality assessment

Table 2 presents the methodological quality assessment of the included studies. Of the 25 studies, 15 were considered to be of high quality (i.e. 5 or more criteria were rated positive). The initial agreement of the total quality assessment was 84% (147 of 175 items). The kappa score calculated on the total quality assessment was 0.65, which is considered to be a substantial agreement. Critical items were 'same duration of follow-up’ and 'follow-up data available from at least 80% of the study population’, which were present in 48% and 52% of the studies, respectively. All initial disagreements were resolved by discussion.

**Table 2:** Quality scores of the included studies

**Authors**	**Criteria**								
	**1**	**2**	**3**	**4**	**5**	**6**	**7**	**Score**	**Quality**
Uziel 2006 [30]	–	–	–	+	–	–	–	1	Low
Skinner 2002 [3]	+	+	+	+	–	+	–	5	High
Kermond 2002 [18]	+	+	+	+	+	+	+	7	High
Fischer 1999 [4]	+	+	+	–	+	+	–	5	High
Mattick 1999 [27]	–	–	+	–	–	+	+	3	Low
Eggl 1999 [12]	+	+	+	+	+	+	–	6	High
De Pellegrin 1997 [11]	+	+	–	+	+	–	–	4	Low
Kesteris 1996 [19]	+	+	+	+	+	–	+	6	High
Keenan 1996 [17]	–	+	–	+	+	+	+	5	High
Taylor 1995 [29]	+	–	+	–	–	–	–	2	Low
Gopakumar 1992 [2]	+	–	+	+	–	–	–	3	Low
Terjesen 1991 [22]	+	+	+	+	+	–	+	6	High
Briggs 1990 [23]	+	–	+	+	–	+	+	5	High
Hasegawa 1988 [14]	+	+	+	–	+	–	+	5	High
Kallio 1988 [16]	+	+	+	–	+	+	+	6	High
Landin 1987 [1]	+	–	+	+	–	–	+	4	Low
Egund 1987 [13]	–	+	+	–	+	+	+	5	High
Kallio 1986 [15]	–	+	+	+	+	+	+	6	High
Haueisen 1986 [24]	+	–	+	+	–	+	–	4	Low
Mukamel 1985 [20]	+	+	+	+	+	–	–	5	High
Illingworth 1983 [25]	+	–	–	–	–	–	–	1	Low
Sharwood 1981 [28]	–	–	–	–	–	–	+	1	Low
Calver 1981 [10]	+	+	+	+	+	+	+	7	High
Mallet 1981 [26]	+	–	+	+	–	–	+	4	Low
Stock 1977 [21]	+	+	–	+	–	–	+	4	Low

+ the criterion was satisfied; – the criterion was not satisfied or it was unclear. Quality criteria: 1) Sample definition given (at least 4 of: age, male–female ratio, in-and exclusion criteria, duration of symptoms prior to submission, diagnostic criteria for transient synovitis), 2) Prospective design, 3) Inception cohort 4) Treatment subsequent to inclusion in cohort is described, 5) Follow-up data available from at least 80% of study population, or reason for loss of follow-up is known, 6) Same duration of follow-up for all patients (maximum range of 30%), or if patients have been followed by a protocol that defines end of follow-up, 7) Same tests used for all patients with transient synovitis during follow-up.

### Synthesis

#### Short-term symptomatic course

Eleven studies [3,11,12,18,21-24,27-29] described the short-term course of TS, of which five are of high quality and six of low quality. The outcomes are shown in Additional file 2: Table S2.

**Figure 2:**
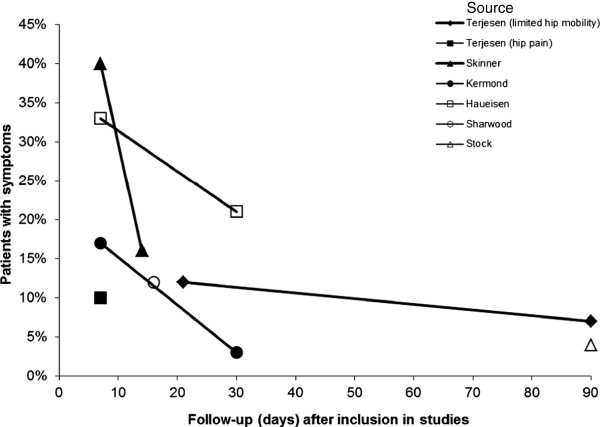
Data on short-term symptoms reported in six studies arranged by high (black) and low (white) methodological quality of the studies.

An overview of six studies [3,18,21,22,24,28], which exclusively reported the percentage of patients with symptoms on short-term follow-up, is shown in Figure 2. Of these, three are of high quality and three of low quality.

In the high quality studies, the length of symptoms (hip pain, limp, limited range of motion and/or refusal to bear weight), ranged from 2.5 days to 3 months [3,12,18,22]. In three of these studies [12,18,22], between 3-8% of patients had symptoms persisting for over one month.

In one high quality study [12], all nine patients with persisting symptoms for over one month developed Legg-Perthes’ disease*.*

In the low quality studies, the length of symptoms ranged from 1 week to 3 months [21,24,28]. Between 4-12% of patients had persisting symptoms for over one month [21,24,28].

#### Recurrence

Thirteen studies [1,2,16,20,22-30] described recurrences of patients with TS (Additional file 2: Table S2). Of these, four are of high quality and nine of low quality.

**Figure 3:**
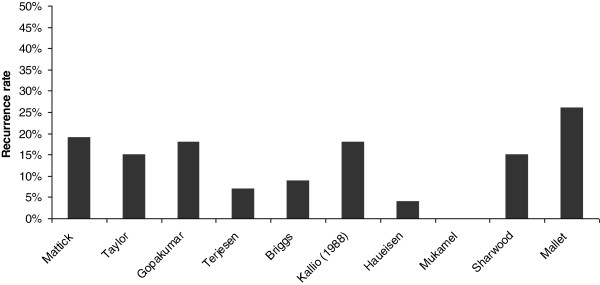
Recurrence rate reported in ten studies.

All four high quality studies [16,20,22,23] calculated a recurrence rate, ranging from 0%^20^ to 18.3%^16^ in a follow-up period ranging from 3 months to 15 years (Figure 3).

According to one high quality study by Terjesen *et al*. [22], 75% of the recurrences occur in the first six months after the initial episode of TS. Also, in 25% of the cases in this study the opposite hip was involved at recurrence.

The recurrence rate was calculated in six low quality studies [2,24,26-29], varying from 4.1% [24] to 26.3% [26] in a follow-up period ranging from 6 months to 20 years.

According to five low quality studies [2,25,26,29,30], recurrences usually appear within the first two years after the initial episode of TS. Five low quality studies [24-26,29,30] showed that in 20-50% of the cases, the opposite hip is involved at recurrence.

#### Long-term course

Nineteen studies [1,2,4,10,13-17,23,24,26-30] observed the long-term course of TS (Additional file 2: Table S2). Of these, ten are of high quality and nine of low quality.

Three high quality studies reported clinical follow-up of TS on the long-term. Limited range of motion was found in 7% to 8% of the patients with TS [15,16].

Ten high quality studies [4,10,13-17,19,20,23] reported development of Legg-Perthes’ disease subsequent to TS, ranging from 0% [15,16,19] to 38% [17]. In a study by by Kallio *et al.*[15] two patients with TS in one hip had recent symptom-free Legg-Perthes’ disease in the opposite hip. Also, in this study all patients initially diagnosed with TS who developed Legg-Perthes’ disease on long-term follow-up had early signs of Legg-Perthes’ disease on the original radiographs. Early signs of Legg-Perthes’ disease on radiographs were defined as signs of avascular necrosis and/or increased joint space.

Two high quality studies mentioned the long-term prevalence of coxa magna subsequent to TS ranging from 0%^23^ to 32% [16].

Five low quality studies reported clinical follow-up of TS on the long-term. Hip pain after intensive physical effort ranged from 12% to 28% [21,26,30]. Limited range of motion of the hip was found in 0% to 18% of the patients with TS [21,28].

Seven low quality studies [1,2,21,24,28-30] reported development of Legg-Perthes’ disease subsequent to TS, ranging from 0% [21,30] to 4% [1].

Two low quality studies reported that patients initially diagnosed with TS who developed Legg-Perthes’ disease on long-term follow-up, had early signs of Legg-Perthes’ disease on the initial radiographs in 67% [2] and 80% [1] of the cases.

## Discussion

As no prognostic review of TS has yet been published, this systematic review is the first to provide an overview of the short and long-term clinical course of children with TS. Both high and low quality studies show that most children with TS experience symptoms for less than two weeks. Recurrence ranged from 0% to 18.3% in the high quality studies and from 4.1% to 26.3% in the low quality studies. In 20% to 50% of the children with a second episode of TS the opposite hip is affected. On long-term follow-up of high quality studies, limited range of motion was found in 7% to 8% of children with TS. Legg-Perthes’ disease subsequent to TS, which was reported most frequently on long-term follow-up, ranged from 0% to 38% in the high-quality studies and from 0% to 4% in the low-quality studies.

Although all the studies followed patients with TS over time their objective(s) sometimes differed, leading to heterogeneity and adversely affecting comparability. With 40% of the included studies having a retrospective design, selection bias may have interfered with the overall outcomes. All included studies described children with TS who were hospitalised or treated as outpatients, none of the patients were seen by a GP only. This might imply that potentially useful information is lost, since many children with TS will be followed only in primary care. Also noteworthy is that the follow-up of children with TS began from the moment of inclusion in the study. This means that the numbers of symptomatic days prior to inclusion are not included in the follow-up period, which might have led to underestimation of the duration of symptoms on short-term follow-up.

The scoring system we used for methodological quality assessment was adopted from an earlier review on assessing clinical course [8], but has not yet been validated. Furthermore, a distinction into high and low quality studies may be too black-and-white a representation of the quality of the included studies. Nevertheless, according to our rating, the methodological quality was high for more than 50% of the included studies. Critical items were criteria 5 and 6 ('same length of follow-up’ and 'follow-up available from at least 80% of the study population’). Both these criteria induced loss of patient information during follow-up and might have influenced the outcomes of the present review. It showed that even within high quality studies large differences in outcomes were seen. The percentage of children with persistent symptoms after one to three weeks of follow-up in Figure 2 ranged from 10% to 40%. The studies^,^ that showed the extreme values of 33% [24] and of 40% [3] did neither use the same test for all patients nor did they have a follow-up larger than 80%. The other studies showed a more homogeneous percentage of 10-17% for persistent symptoms after one to three weeks of follow-up.

For recurrence rate the largest differences was 0% after 9.3 months [20] and 19% after one year [16]. In this last study an extensive systematic re-examination in all patients was used, while the other study re-examined only some of the patients at three months based on initial findings and just reported that there was no recurrence between three months and three years of follow-up. A low quality study also showed a large recurrence rate after seven months of follow-up, but this study was retrospective and had more than 20% loss to follow-up [2].

The relatively high recurrence rate of TS at the opposite hip supports the hypothesis that TS is due to a systemic factor rather than being a local problem. A review by Do [5] includes various studies suggesting that a viral agent (causing respiratory tract infection or other flu-like syndromes) may also lead to the development of TS in children; elevated viral titers have been found in children with TS, although an infectious agent has not yet been identified.

The prevalence of Legg-Perthes’ disease subsequent to TS (reported in nine high-quality studies) [4,10,13-16,19,20,23] ranged from 0% to 10%. One high-quality study by Keenan *et al.*[17] reported that 38% of the cases initially diagnosed as TS subsequently developed Legg-Perthes’ disease. The aim of this latter study differs from the rest since only 13 patients with either prolonged or recurrent TS were studied. Five of them had a delay of more than two years in bone age and all five developed changes of Legg-Perthes’ disease at three-months follow-up. These outcomes are consistent with the earlier mentioned hypothesis of a higher risk of Legg-Perthes’ disease in children with prolonged symptoms of TS [1]. However, due to the low number of included patients and the preselected group of children with TS, the prevalence of 38% should not be generalized to the risk of developing Legg-Perthes’ disease subsequent to TS.

Gaughan *et al.* describe an estimated prevalence rate of Legg-Perthes’ disease (reported in the general pediatric population in the USA) of 0.023% [31]. The high quality studies in our review report a prevalence of Legg-Perthes’ disease subsequent to TS of 0-10%. The question remains whether Legg-Perthes’ disease is a direct consequence of TS in children. Symptoms of TS could also represent early signs of Legg-Perthes’ disease. Children initially diagnosed with TS and (after ongoing symptoms) subsequently diagnosed with Legg-Perthes’ disease fit this hypothesis. Early signs of Legg-Perthes’ disease found at retrospective analysis of initial radiographs of patients with TS [15], also support this theory. Moreover, subtle radiologic changes of Legg-Perthes’ disease are easily overlooked. In the literature, Gopakumar *et al.* found an absence of early radiologic signs in about 20% of the patients at first presentation, and in about 50% of the patients diagnosed with Legg-Perthes’ disease [2].

Regrettably, some studies excluded children with Legg-Perthes’ disease whereas information about the medical history of these patients might have revealed a misdiagnosis in TS. The reported follow-up periods after the initial diagnosis of TS in which subsequent Legg-Perthes’ was diagnosed ranged between 1 and 6 months [1,10,11,17,20,24,28]. This might indicate that some studies did not follow their patients long enough to detect this.

## Conclusions

The majority of the studies indicate that children with TS recover within two weeks; recurrence was seen in 0-26% of the cases. In the future, a long-term cohort study would be useful to more precisely evaluate the course of TS. Meanwhile, given the knowledge so far, it seems warranted to follow children with TS for at least 6 months to ensure that no hip symptoms are present that might indicate Legg-Perthes’ disease.

## Abbreviations

TS: Transient synovitis of the hip.

## Competing interests

All authors declare no competing of interest. This article was commissioned and peer-reviewed.

## Authors’ contributions

RvR, MK, JB, and SB initiated the study. SA designed and performed the search, and wrote the manuscript. SA and RvR selected relevant articles from the titles and abstracts. SA and SB included final from the selected full text articles. SB, JB, and SA assessed quality, and SA and MK extracted data from the selected articles. All authors were involved in critically summarising the evidence and writing the article, and agreed with the final version.

## Supplementary Material

Additional file 1 Detailed search strategy. Click here for file

Additional file 2 Outcomes of the studies. Click here for file
