# Supplementary material for: What is the clinical course of transient synovitis in children: a systematic review of the literature
Source: Chiropr Man Therap. 2013 Nov 14;21:39. doi: 10.1186/2045-709X-21-39 (PMC3831260; doi:10.1186/2045-709X-21-39)
Supplement: Additional file 1 — Detailed search strategy. [file 2045-709X-21-39-S1.doc]

eTable 1 Details of the search strategy.

|  | Medline (Jan 1966-Sept 2013) | Embase (Jan 1947- Sept 2013) |
| --- | --- | --- |
| Diagnosis | (transient synovitis[tw] OR coxitis fugax[tw] OR irritable hip[tw]) | ('transient synovitis':ti,ab,de OR 'coxitis fugax':ti,ab,de OR 'irritable hip':ti,ab,de) |
| Age group | (infant[mesh] OR infan*[tw] OR child[mesh] OR child*[tw] OR pediatr*[tw] OR paediatr*[tw] OR newborn*[tw] OR neonat*[tw] OR adolescent[mesh] OR adolescen*[tw]) | (childhood/exp OR infan*:ti,ab,de OR child*:ti,ab,de OR pediatr*:ti,ab,de OR paediatr*:ti,ab,de OR newborn*:ti,ab,de OR neonat*:ti,ab,de OR adolescence/syn OR adolescen*:ti,ab,de OR [newborn]/lim OR [infant]/lim OR [preschool]/lim OR [school]/lim OR [child]/lim OR [adolescent]/lim) |
| Outcome | (prognosis[mesh] OR prognosis[tw] OR recurrence[mesh] OR recurrence[tw] OR cohort studies[mesh] OR cohort[tw] OR follow-up[tw] OR followup[tw] OR longitudinal[tw] OR prospective[tw] OR randomized controlled trials as topic[mesh] OR randomized controlled trial[pt] OR randomized controlled trial*[tw] OR case-control studies[mesh] OR case-control*[tw] OR retrospective[tw] OR long term[tw] OR predict*[tw] OR course[tw] OR outcome[tw]) | (prognosis:ti,ab,de OR 'recurrent disease'/syn OR recurrence:ti,ab,de OR 'cohort analysis'/syn OR cohort:ti,ab,de OR 'follow up'/syn OR 'follow-up':ti,ab,de OR followup:ti,ab,de OR 'longitudinal study'/syn OR longitudinal:ti,ab,de OR 'prospective study'/syn OR prospective:ti,ab,de OR 'randomized controlled trial'/syn OR 'randomized controlled trial':ti,ab,de,it OR 'randomized controlled trials':ti,ab,de OR 'case-control study'/syn OR (case NEAR/1 control*):ti,ab,de OR 'retrospective study'/syn OR retrospective:ti,ab,de OR 'long term':ti,ab,de OR predict*:ti,ab,de OR 'disease course'/syn OR course:ti,ab,de OR outcome:ti,ab,de) |
| Combination | *Diagnosis* and *Age group* and *Outcome* | *Diagnosis* and *Age group* and *Outcome* |
