# Supplementary material for: What is the clinical course of transient synovitis in children: a systematic review of the literature
Source: Chiropr Man Therap. 2013 Nov 14;21:39. doi: 10.1186/2045-709X-21-39 (PMC3831260; doi:10.1186/2045-709X-21-39)
Supplement: Additional file 2 — Outcomes of the studies. [file 2045-709X-21-39-S2.doc]

| **Short-term symptomatic course**  **eTable 2 Study Outcomes** | | | | |
| --- | --- | --- | --- | --- |
| ***Study*** | ***Patients^a^*** | ***Follow-up*** | ***Outcomes*** | ***Quality Score*** |
| Haueisen,1986 | n=458 | 1 week  1 month  > 1 month | 33% (n=152) limp or refusal to bear weight  21% (n=99) limp or refusal to bear weight  12% (n=53) limp or refusal to bear weight  *Mean duration of admission to hospital*  6.9 (1-32) days | Low |
| Kermond, 2002 | n=36 | 7 days  1 month | 17% (n=6) hip pain, limp or both  3% (n=1) hip pain, limp or both  *Mean duration of symptoms*  2.5 days (ibuprofen) and 4.5 days (placebo) | High |
| Sharwood, 1981 | n=101 | Unclear | Symptoms settled in most cases within 16 days and  lasted more than twice the average time in 12%  (n=12), of which 1 case subsequently developed  Legg-Perthes disease and 1 coxa magna | Low |
| Skinner, 2002 | n=25  n=10 | 7 days  14 days | 40% (n=10) abnormal clinical exam with effusion on ultrasound    16% (n=4) persisting effusion on ultrasound but symptom free  *Median duration until clinical resolution or pain free*  4 (3-12) days | High |
| Stock, 1977 | n=26 | 3 months | 4% (n=1) hip pain | Low |
| Terjesen, 1991 | n=58 | 1 week  3 weeks  3 months  6 weeks | 10% (n=6) hip pain  12% (n=7) limited ROM^b^  7% (n=4) limited ROM  1.7% (n=1) Legg-Perthes’ disease | High |
| Eggl, 1999 | n=106 | 6 weeks | 8% (n=9) hip pain + limited ROM, all subsequently  diagnosed with Legg-Perthes’ disease  *Mean duration of pain*  9 days  *Mean duration of limited ROM*  11 days | High |
| De Pellegrin, 1997 | n=100 | 4-6 weeks | 3% (n=3) Legg-Perthes’ disease | Low |
| Mattick 1999 | n=33 | 7 years | *Mean duration of admission to hospital*  3 (1-8) days | Low |
| Taylor 1995 | n=358  n=42  n=11 | Unclear | *Mean duration of admission to hospital*  3.7 days (first admission)  4.2 days (second admission)  4.9 days (> 2 admissions) | Low |
| Briggs 1990 | n=286^c^ | Unclear | *Mean duration of admission to hospital*  5.9 ± 2.96 days (no traction of the hip)  6.43 ± 2.96 days (traction of the hip) | High |
| **Recurrence** | | | | |
| ***Study*** | ***Patients^a^*** | ***Follow-up*** | ***Outcomes*** | ***Quality Score*** |
| Mattick, 1999 | n=73 | 7 years | *Recurrence*  19.2% (n=14) | Low |
| Taylor, 1995 | n=358 | Unclear | *Recurrence*  14.8% (n=53)  *Opposite hip at recurrence*  42% (n=18)  *Onset of recurrence*  < 1 year after initial episode:  51% (n=27) | Low |
| Gopakumar, 1992 | n=181 | 7 (6-24) months | *Recurrence*  18.2% (n=33)  *Onset of recurrence*  < 1 year after initial episode: 54% (n=18) | Low |
| Terjesen, 1991 | n=58 | 5.8 (2-15) years | *Recurrence*  6.9% (n=4)  *Opposite hip at recurrence*  25% (n=1)  *Onset of recurrence*  < 6 months after initial episode: 75% (n=3)  2 years after initial episode: 25% (n=1) | High |
| Briggs, 1990 | n=286 | Unclear | *Recurrence*  9.4% (n=27) | High |
| Kallio, 1988 | n=109 | 1 year | *Recurrence*  18.3% (n=20) | High |
| Haueisen, 1986 | n=458 | Unclear | *Recurrence*  4.1% (n=19)  *Opposite hip at recurrence*  26% (n=5)  *Interval between episodes*  1 year (2 weeks-4 years) | Low |
| Mukamel, 1985 | n=41 | 9.3 months  (3 months-3 years) | *Recurrence*  0.0% (n=0) | High |
| Sharwood, 1981 | n=101 | 8.2 (5-15) years | *Recurrence*  14.9% (n=15) | Low |
| Mallet, 1981 | n=38 | 7 (2-20) years | *Recurrence*  26.3% (n=10)  *Opposite hip at recurrence*  20% (n=2)  *Onset of recurrence^d^*  < 1 year after initial episode: 38% (n=5)  < 2 years after initial episode: 15% (n=2)  4 years after initial episode: 8% (n=1)  7 years after initial episode: 8% (n=1) | Low |
| Landin, 1987 | n=258 | 5 years | *Risk of recurrence^e^*  20:1 | Low |
| Illingworth, 1983 | n=54  n=70^f^ | Unclear | *Opposite hip at recurrence*  ± 50% (n not given)  *Onset of recurrence*  < 6 months after initial episode: 43% (n=30)  7-12 months after initial episode: 29% (n=20)  During second year: 13% (n=9)  > 2 years: 15% (n=11) | Low |
| Uziel, 2006 | n=39 | 4.2 ± 2.5 years | *Opposite hip at recurrence*  36% (n=14)  *Onset of recurrence*  < 1 year after initial episode: 69% (n=27)  During second year: 13% (n=5)  > 2 years: 18% (n=7) | Low |
| **Long-term course** | | | | |
| ***Study*** | ***Patients^a^*** | ***Follow-up*** | ***Outcome*** | ***Quality Score*** |
| Uziel, 2006 | n=39 | 4.2 ± 2.5 years | *Development into other diseases*  Spondylarthropathy 5% (n=2)  Rheumatic diseases 8% (n=3)  FMF^g^ 3% (n=1)  Legg-Perthes’ disease or other chronic orthopaedic  conditions 0%  *Clinical follow-up*  Hip pain after intensive physical effort 28% (n=10)  Episodes of limping 14% (n=5) | Low |
| Fischer, 1999 | n=65 | 18-21 months | *Development into other diseases*  Legg-Perthes’ disease 2% (n not given) | High |
| Mattick, 1999 | n=79 | 7 years | No long-term complications^h^ | Low |
| Kesteris, 1996 | n=21 | 6-12 months | *Development into other diseases*  Legg-Perthes’ disease 0% | High |
| Keenan, 1996 | n=13 | 3 months | *Development into other diseases*  Legg**-**Perthes’ disease 38% (n=5) | High |
| Taylor, 1995 | n=358 | Unclear | *Development into other diseases*  Legg-Perthes’ disease 0.3% (n=1) | Low |
| Gopakumar, 1992 | n=181 | 7 (6-24) months | *Development into other diseases*  Legg-Perthes’ disease 2% (n=4)  Septic arthritis 2% (n=3) | Low |
| Briggs, 1990 | n=286 | Unclear | *Development into other diseases*  Legg-Perthes’ disease 0.3% (n=1)  Coxa magna or other abnormality in the hip joint  0% | High |
| Hasegawa, 1988 | n=55^h^ | 4-9 months | *Development into other diseases*  Legg-Perthes’ disease 2% (n=1) | High |
| Kallio, 1988 | n=109 | 1 year | *Development into other diseases*  Coxa magna 32% (n=35)  Legg-Perthes’ disease 0%  *Clinical follow-up*  Limited ROM 7% (n=8) | High |
| Landin, 1987 | n=258 | 5 years | *Development into other diseases*  Legg-Perthes’ disease 4% (n=10)  Rheumatic diseases 0.3% (n=2)  *Clinical follow-up*  No symptoms on long-term follow-up | Low |
| Egund, 1987 | n=49 | 6.5 (5-9) months | *Development into other diseases*  Legg-Perthes’ disease 2% (n=1)  *Clinical follow-up*  No symptoms on long-term follow-up | High |
| Kallio, 1986 | n=119 | 1 year | *Development into other diseases*  Legg-Perthes’ disease 0%  *Clinical follow-up*  Limited ROM 8% (n=8) | High |
| Haueisen, 1986 | n=118 | 6 months | *Development into other diseases*  Legg-Perthes’ disease 3% (n=3)  Rheumatic diseases 2% (n=2)  Osteoid osteoma 0.8% (n=1)  Coxa magna 0% | Low |
| Mukamel, 1985 | n=41 | 9.3 months (3 months-3 years) | *Development into other diseases*  Legg-Perthes’ disease 1% (n=1)^j^ | High |
| Sharwood, 1981 | n=101 | 8.2 (5-15) years | *Development into other diseases*  Legg-Perthes’ disease 1% (n=1)  Coxa magna 1% (n=1)  Slipped upper femoral epiphysis 2% (n=2)  *Clinical follow-up*  Limited ROM 18% (n=18)  Intermittent pain in hip joint 8% (n=8) | Low |
| Mallet, 1981 | n=38 | 7 (2-20) years | *Development into other diseases*  Coxa magna 11% (n=4)  *Clinical follow-up*  Hip pain after intensive physical effort 18% (n=7) | Low |
| Calver, 1981 | n=50 | 1 year | *Development into other diseases*  Legg-Perthes’ disease 10% (n=5) | High |
| Stock, 1977 | n=26 | 4.3 (0.5-15) years | *Development into other diseases*  Legg-Perthes’ disease 0%  *Clinical follow-up*  Hip pain after intensive physical effort 12% (n=3)  ROM of the hip in all patients normal | Low |
| *^a^ Number of patients after loss to follow-up; ^b^ Range of motion of the hip; ^c^ Numbers of patients treated with or without traction are not given; ^d^ Risk of recurrence of TS compared to the risk of a first episode of TS; ^e^ Calculated with number of recurrences of hips (n=13); ^f^ Number of recurrences; ^g^ Familial Mediterranean fever; ^h^ Unclear which diseases have been investigated; ^i^ Initially 56 patients with clinical TS, one excluded because of development into Legg-Perthes’ disease; ^j^ One of ten patients diagnosed with Legg-Perthes’ disease was initially diagnosed with TS* | | | | |
